# Symmetry-Induced Emergence of a Pseudo-Qutrit in the Dipolar Coupling of Two Qubits

**DOI:** 10.3390/e24020223

**Published:** 2022-01-31

**Authors:** Yury Belousov, Vladimir I. Man’ko, Agostino Migliore, Alessandro Sergi, Antonino Messina

**Affiliations:** 1 Moscow Institute of Physics and Technology, Institutskii Per. 9, Dolgoprudny, 141700 Moscow, Russia; belousov.yum@phystech.edu; 2 Terra Quantum AG, St. Gallerstrasse 16A, 9400 Rorschach, Switzerland; 3 Lebedev Physical Institute, Leninskii Prospect 53, 119991 Moscow, Russia; manko@sci.lebedev.ru; 4 Department of Chemical Sciences, University of Padova, Via Marzolo 1, 35131 Padova, Italy; agostino.migliore@unipd.it; 5 Dipartimento di Scienze Matematiche e Informatiche, Scienze Fisiche e Scienze della Terra, Università degli Studi di Messina, Viale F. Stagno d’Alcontres 31, 98166 Messina, Italy; asergi@unime.it; 6 Istituto Nazionale di Fisica Nucleare, Sez. di Catania, 95123 Catania, Italy; 7 Institute of Systems Science, Durban University of Technology, P.O. Box 1334, Durban 4000, South Africa; 8 Dipartimento di Matematica ed Informatica dell’Università di Palermo, Via Archirafi 34, 90123 Palermo, Italy

**Keywords:** two-spin systems, hyperfine interactions, symmetry-constrained dynamics, qutrit, density matrix, unitary transformation, spin polarization, probability representation, 03.65.-w, 67.57.Lm, 74.20.Mn, 81P99, 81Q99, 82D40

## Abstract

We investigate a system of two identical and distinguishable spins 1/2, with a direct magnetic dipole–dipole interaction, in an external magnetic field. Constraining the hyperfine tensor to exhibit axial symmetry generates the notable symmetry properties of the corresponding Hamiltonian model. In fact, we show that the reduction of the anisotropy induces the invariance of the Hamiltonian in the 3×3 subspace of the Hilbert space of the two spins in which ${\hat{S}}^{2}$ invariably assumes its highest eigenvalue of 2. By means of appropriate mapping, it is then possible to choose initial density matrices of the two-spin system that evolve in such a way as to exactly simulate the time evolution of a pseudo-qutrit, in the sense that the the actual two-spin system nests the subdynamics of a qutrit regardless of the strength of the magnetic field. The occurrence of this dynamic similitude is investigated using two types of representation for the initial density matrix of the two spins. We show that the qutrit state emerges when the initial polarizations and probability vectors of the two spins are equal to each other. Further restrictions on the components of the probability vectors are reported and discussed.

## 1. Introduction

The properties of systems of few spins are a classic problem in physics and are the subject of growing interest in the scientific community because of their potential for applications in quantum information theory and related quantum computing technologies [1,2,3]. In fact, spin s = 1/2 two-level systems are the prototype qubit and therefore the most basic unit of quantum information. The new routes to using spin systems in such fields of investigations [4,5,6,7,8] have raised a vast gamut of physical problems which have not been considered in standard NMR and ESR applications of spin systems and their hyperfine interactions.

There is, in particular, a renewed interest in the dynamic problem of two interacting identical spins as considered from the perspective of quantum information technology, which differs from the statement of the problem in traditional NMR research. NMR is generally used at the limit of high external magnetic fields. Then, the nuclear magnetic dipole–dipole interaction is treated as a small perturbation. This small perturbation can give very interesting effects, as has been shown in studies of paramagnetic radicals and other magnetic systems more complex than two interacting identical nuclear spins. On the contrary, results of interest to the field of quantum information, such as the preparation of entangled states of two nuclear spins, can be obtained when the external magnetic fields are small compared to the nuclear magnetic fields.

It is interesting to tackle this problem first for a system of two interacting and neighboring identical nuclear spins in a crystal lattice. We assume that their spatial configuration is such that their physical properties are experimentally accessible individually. For this reason, the consequences of identity can be ignored when investigating their dynamics [9]. In other words, the two particles are identical (that is, they have the same intrinsic properties, such as electric charge, spin, etc., which do not depend on the state of the system) but are distinguishable (namely, they differ by their extrinsic properties, such as the spatial distribution, which depend on the system state) [10]. The more frequently used nuclear isotopes with nonzero spin I=1/2 are ${}^{13}$C, with a magnetic moment $\mu_{C}=+0.70238\mu_{n}$ (and a natural occurrence of 1.108%), and ${}^{15}$N with $\mu_{N}=-0.283049\mu_{n}$ (0.365%). Clearly, we must not forget the main nuclear hydrogen isotope ${}^{1}$H, with $\mu_{p}=+2.79867\mu_{n}$, where the nuclear magneton is given by $\mu_{n}=5.05095\times 10^{-24}$Erg/G. Isotopes ${}^{13}$C and ${}^{15}$N are very interesting because they are the main impurities in diamond crystals of great relevance to quantum technologies. Silicon has a diamond cubic crystal structure and is the second most popular material for quantum technologies. Isotope ${}^{29}$Si, with a nonzero nuclear spin of I=1/2, has a magnetic moment of $\mu_{Si}=-0.55\mu_{n}$ and a natural occurrence of 4.67%.

Useful physical examples of two interacting identical spins are provided by “frozen” diatomic molecules with identical nuclei (e.g., the hydrogen molecule H${}_{2}$ at sufficiently low, ideally zero, temperature), or approximately, by a pair of adjacent protons or other identical isotopes in more complicated molecules. Such molecules should be frozen in order to exclude the averaging of the direct nuclear magnetic dipole–dipole interaction over the different rotational states of the molecule. In this limit, there is no connection between the spin and rotational states of the nuclei due to the Pauli principle.

Therefore, it is interesting to solve the physical problem of two interacting identical nuclear spins that are nearest neighbors in a crystal lattice (at zero or a sufficiently low temperature) or in some other “frozen” configuration. In standard quantum mechanics, the states of quantum systems are described by means of the corresponding wave functions [11], vectors in a Hilbert space [12], density matrices [13], and density operators [14]. Recently, the probability representation of quantum states was constructed [15,16], where spin states are described by standard classical probability distributions [17,18,19]. This approach has not yet been used to study systems of two identical 1/2 spins. We also note that some years ago, a partial probability representation of a spin system was framed in an algebraic form [20]. To date, the probability representation has been used to study the effects of thermal fluctuations on the dynamics of a single spin [21,22,23] and of a spin-chain [24] of minimal length. Considering the important role of spin systems in quantum information and computing, as well as in related technologies, in this article, we study the system of two fermions with spin 1/2, describing it in terms of both spin polarization and probability distribution.

The paper is organized as follows. Section 2 is devoted to the construction of the Hamiltonian model that describes the physical scenario under investigation. The systematic search for symmetries and constants of motion exhibited by the Hamiltonian model is reported in Section 3. A similar route has recently been used to investigate the dynamics of a pair [25,26,27,28,29,30,31] or a chain [32,33] of coupled spins (also greater than $\frac{1}{2}$) subjected to time-independent and time-dependent [34,35,36] external magnetic fields. Symmetry arguments have also been exploited to elegantly bring to light intriguing dynamic features of physical systems living in Hilbert spaces of infinite dimensions [37,38,39,40,41,42,43,44,45,46,47,48,49,50,51,52,53,54,55,56,57,58,59,60,61]. In Section 4 it is demonstrated that the time evolution of the two dipolarly coupled spins nests a qutrit subdynamics governed by a Hamiltonian model that is explicitly derived. This is the main result achieved in the paper.

The expected consequent interrelations between the density matrix of the two coupled spins and that of the emerging qutrit are studied in Section 6 and Section 7, where we use representations of the generic density matrix of the binary system that are based on polarization vectors or on the use of classical probabilities. Concluding remarks follow in Section 8.

## 2. The Hamiltonian Model

Our physical model consists of two interacting and identical nuclear spins 1/2. The two nuclei are distinguishable since they are localized at two neighboring fixed points of a crystal lattice separated by the vector R=Rn, n being a unit vector and *R* the distance between the two nuclei.

An external, uniform, and static magnetic field B, oriented along the z-axis of a Cartesian laboratory frame, acts on the two magnetic nuclear dipoles $g\mu_{0}{\hat{s}}_{1}$ and $g\mu_{0}{\hat{s}}_{2}$, *g* and $\mu_{0}$ being the appropriate nuclear *g*-factor and magneton, respectively. The coupling between the two spins originates from the dipole–dipole interaction. Similarly to the classical expression, it depends on the relative orientations of the two spins and is therefore anisotropic.

The Hamiltonian model governing the dynamics of the system of two separated identical nuclear spins 1/2 in its Hilbert space H can generally be written as follows:

(1) $$\hat{H}=-\omega \left({\hat{s}}_{1z}+{\hat{s}}_{2z}\right)-3\Omega \left(n{\hat{s}}_{1}\right)\left(n{\hat{s}}_{2}\right)+\Omega \left({\hat{s}}_{1}{\hat{s}}_{2}\right),$$

where $\omega =g\mu_{0}B$, $\Omega ={\left(g\mu_{0}\right)}^{2}/R^{3}$ is the dipole coupling strength, and the energy is expressed in frequency units, i.e., ℏ=1.

H is the tensorial product of $H_{1}$ and $H_{2}$, i.e., the Hilbert spaces of the first and the second spin, respectively. The standard basis in H is the following ordered direct product of the conventional bases in $H_{1}$ and $H_{2}$:

(2) |+〉|+〉,|+〉|−〉,|−〉|+〉,|−〉|−〉,

where the first (second) ket in each product is one of the two possible eigenstates of ${\hat{s}}_{1z}$(${\hat{s}}_{2z}$) corresponding to the eigenvalues ±1/2.

It is useful to express $\hat{H}$ in terms of the Cartesian components of the vector operators ${\hat{s}}_{1}$ and ${\hat{s}}_{2}$, that is $\left({\hat{s}}_{1x},{\hat{s}}_{1y},{\hat{s}}_{1z}\right)$ and $\left({\hat{s}}_{2x},{\hat{s}}_{2y},{\hat{s}}_{2z}\right)$, respectively:

(3) $$\hat{H}=-\omega \left({\hat{s}}_{1z}+{\hat{s}}_{2z}\right)+A_{ik}{\hat{s}}_{1i}{\hat{s}}_{2k}.$$

The nine coefficients $A_{ik}\left(i,k=1,2,3\right)$ are the matrix elements of the second-rank Cartesian and orthogonal tensor A, which incorporates all the features of the hyperfine interaction. Comparing the spin–spin interaction term in the Hamiltonian (3) with the expression for the dipole–dipole interaction in (1), we get the following equation:

(4) $$A_{ik}=-\Omega \left(3n_{i}n_{k}-\delta_{ik}\right)=A_{ki},$$

where $\left(n_{1},n_{2},n_{3}\right)$ are the components of the unit vector n.

The previous expression (4) shows that the elements of A are proportional to Ω and generally depend on two further parameters that identify n.

Despite the arbitrariness of n, the Hamiltonian (1) commutes with the square of the total angular momentum, ${\hat{S}}^{2}$, where $\hat{S}={\hat{s}}_{1}+{\hat{s}}_{2}$. It is worth noting that the three-dimensional eigen-subspace of ${\hat{S}}^{2}$ pertaining to its eigenvalue S=2 is dynamically invariant, that is, it is invariant under $\hat{H}$ application. This property spurs us toward our goal of revealing physical situations in which the dipolar coupling nests the dynamics of a qutrit. Unfortunately, making such behavior evident in a general scenario (i.e., for an arbitray n) requires tedious and distracting algebraic manipulations. For this reason, an intermediate goal of our project is to select sub-models of the Hamiltonian (3) that have a higher level of symmetry than that possessed by the Hamiltonian (3). The key idea is to focus on the tensor A, searching for conditions on n that make it axial symmetric. The corresponding Hamiltonian model will depend on few parameters and will clearly still be ${\hat{S}}^{2}$-conserving. We point out that if the hyperfine tensor were spherically symmetric, then A would be proportional to the identity matrix (it is useful to remember that A is diagonalizable), thus stripping the corresponding Hamiltonian of physical interest for the scope of this study.

To impose axial symmetry on A, it is sufficient to require that A admits an eigenvalue $\varepsilon_{0}\neq 0$ of multiplicity 2 and a third eigenvalue equal to $\alpha \varepsilon_{0}$, with α≠0 ($\varepsilon_{0}$ has the same dimension as Ω since ℏ=1).

Forcing the roots of the characteristic equation of A to be $\varepsilon_{0}$, $\varepsilon_{0}$, and $\alpha \varepsilon_{0}$ leads to the following nonlinear system in the six unknown elements of A:

(5) $$\left\{\begin{array}{c} A_{xx}+A_{yy}+A_{zz}=\left(\alpha +2\right)\varepsilon_{0}\\ (A_{xx}A_{yy}-\left(A_{xy}^{2}\right)+\left(A_{xx}A_{zz}-{\left(A_{xz}\right)}^{2}\right)+\left(A_{yy}A_{zz}-{\left(A_{yz}\right)}^{2}\right)=\left(2\alpha +1\right)\varepsilon_{0}^{2}\\ detA=\alpha \varepsilon_{0}^{3} \end{array}\right.$$

where we used the compact notation detA rather than writing the full expression for the determinant of A in terms of its elements.

We remark that our intermediate goal is to find at least one solution for this system that is compatible with the link between A and the components of n defined in (4).

We therefore simplify the algebraic problem (practically reducing the number of unknowns) by looking for a solution that makes A an X matrix (an X matrix is defined by the condition that all its elements out of the principal and secondary diagonals vanish) [62], that is, by imposing the necessary conditions $\;A_{xy}=A_{yz}=0$ and hence $\;A_{yx}=A_{zy}=0$. In view of (4), this choice implies the restriction $n_{2}=0$, which means that n is parallel to the xz coordinate plane. The first advantage of this choice is that the second equation of system (5) is satisfied by the solutions of the following symmetric system:

(6) $$\left\{\begin{array}{c} A_{xx}+A_{zz}=\left(\alpha +1\right)\varepsilon_{0}\\ A_{xx}A_{zz}=\alpha \varepsilon_{0}^{2}+{\left(A_{xz}\right)}^{2}, \end{array}\right.$$

where $\;A_{xz}$ is firstly treated as a parameter. Since $\;A_{xx}$ and $\;A_{zz}$ are real, $\;A_{xz}$ must satisfy the following condition:

(7) $$|A_{xz}|\leq \frac{\left|\alpha -1|\right|\varepsilon_{0}|}{2},$$

which leads us to fix

(8) $$A_{xz}=\frac{\left(\alpha -1\right)\varepsilon_{0}}{2}\sin2\theta ,$$

where θ can vary in the range [0, π). Since, according to definition (4), $A_{xz}$ is proportional to Ω, and α is independent of Ω, we must fix $\varepsilon_{0}=\Omega$ for any model that we may extract from the Hamiltonian (1).

We can now easily write the two solutions of system (6) in the following form:

(9) $$\left\{\begin{array}{c} A_{xx}\vee A_{zz}=\left(\left(\alpha -1\right)\cos^{2}\theta +1\right)\varepsilon_{0}\\ A_{zz}\vee A_{xx}=\left(\left(\alpha -1\right)\sin^{2}\theta +1\right)\varepsilon_{0}. \end{array}\right.$$

where, as in set theory, ∨ stands for the logical or. The interchangeability of the expressions for $A_{xx}$ and $A_{zz}$ is a result of the symmetry properties of the system.

The normalized eigenvector $u_{\alpha }$ of A corresponding to its eigenvalue $\alpha \varepsilon_{0}$ fixes the direction of the hyperfine tensor axis, and for the first solution in Equation (9), it can be written as follows:

(10) $$u_{\alpha }^{T}=\left(\cos\theta ,0,\sin\theta \right),$$

The second solution in Equation (9) gives instead

(11) $$u_{\alpha }^{T}=\left(\sin\theta ,0,\cos\theta \right),$$

where the superscript *T* denotes the transposition. Therefore, the parameter θ, which results naturally from our construction, represents the angle between the *x*-axis (the *z*-axis) of the laboratory frame and the symmetry axis $u_{\alpha }$ of the hyperfine interaction tensor when the first (the second) solution (9) of the system (6) is chosen. We underline that both choices are legitimate and generate different physical scenarios corresponding to different Hamiltonian models.

So far, no restrictions have been imposed on α, except for α≠0,1. Therefore, to complete the construction of a specific Hamiltonian model extracted from the general Hamiltonian model (1) in which A exhibits axial symmetry, we need to fix α so that the solutions found for $A_{xx}$, $A_{zz}$,$A_{xz}$ are compatible with (4). It is possible to show that α=−2 is the only value compatible with such requirement.

Finally, by selecting the second solution of the system (6), A can be cast in the following form:

(12) $$A=\left(\begin{array}{ccc} \Omega (1-3\sin^{2}\theta ) & 0 & \frac{-3\Omega }{2}\sin2\theta \\ 0 & \Omega & 0\\ \frac{-3\Omega }{2}\sin2\theta & 0 & \Omega (1-3\cos^{2}\theta ) \end{array}\right)$$

Ultimately, our analysis produces the Hamiltonian model

(13) $$\begin{array}{ccc} {\hat{H}}_{as} & = & -\omega \left({\hat{s}}_{1z}+{\hat{s}}_{2z}\right)+\Omega \left(1-3\sin^{2}\theta \right){\hat{s}}_{1x}{\hat{s}}_{2x}+\Omega {\hat{s}}_{1y}{\hat{s}}_{2y}+\Omega \left(1-3\cos^{2}\theta \right){\hat{s}}_{1z}{\hat{s}}_{2z}\\ & & +\frac{-3\Omega }{2}\sin2\theta \left({\hat{s}}_{1z}{\hat{s}}_{2x}+{\hat{s}}_{1x}{\hat{s}}_{2z}\right), \end{array}$$

which describes two identical but distinguishable spin-1/2 particles that are subject to a static and uniform external magnetic field parallel to the *z*-axis and to an internal, anisotropic dipolar interaction with axial symmetry around a direction that forms an angle θ with the *z*-axis of the laboratory frame and hence with the external magnetic field. It is easy to see that by setting $n=(n_{1}=\sin\theta ,n_{2}=0,n_{3}=\cos\theta )$ in the Hamiltonian (1), one obtains ${\hat{H}}_{as}$. Thus, n is parallel to the symmetry axis of A. We emphasize that the Hamiltonian (13) is not an approximate toy model surrogate of the Hamiltonian (1). Rather, by specializing n in (1), ${\hat{H}}_{as}$ describes an exemplary class of ${\hat{S}}^{2}$-conserving submodels of (1) that only depend on three parameters (instead of four) as a result of the axial symmetry imposed on the X matrix-shaped A tensor. These symmetry properties and the related physical consequences will be discussed in detail in the next section.

Upon direct inspection of Equation (13), it can be easily seen that $tr{\hat{H}}_{as}=0$. Furthermore, since ${\hat{H}}_{as}$ has the matrix representation

(14) $${\hat{H}}_{as}=\frac{\Omega }{8}\left(\begin{array}{cccc} 2(1-3\cos^{2}\theta )-\omega & -6\sin^{2}\theta & -3\sin2\theta & -3\sin2\theta \\ -6\sin^{2}\theta & 2(1-3\cos^{2}\theta )+\omega & 3\sin2\theta & 3\sin2\theta \\ -3\sin2\theta & 3\sin2\theta & -2(1-3\cos^{2}\theta ) & 2(2-3\sin^{2}\theta )\\ -3\sin2\theta & 3\sin2\theta & 2(2-3\sin^{2}\theta ) & -2(1-3\cos^{2}\theta ) \end{array}\right)$$

in the ordered basis (which, as an ordered set, is a permutation of the basis in (2))

(15) $$|\chi_{1}\rangle =|+\rangle |+\rangle ,\;|\chi_{2}\rangle =|-\rangle |-\rangle ,\;|\chi_{3}\rangle =|-\rangle |+\rangle ,\;|\chi_{4}\rangle =|+\rangle |-\rangle ,$$

the two equal rows in (14) imply that it is also $det{\hat{H}}_{as}=0$. Consequently, one of the eigenvalues of ${\hat{H}}_{as}$ vanishes whatever the value of θ. Moreover, since the presence of ω implies that the first three rows of ${\hat{H}}_{as}$ are linearly independent, the null eigenvalue must be a singlet.

The analysis that we will develop in the next sections is based on ${\hat{H}}_{as}$. We will start by showing the symmetry properties of ${\hat{H}}_{as}$ and how they lead to the emergence of a three-dimensional subspace of H in which the time evolution of the two spins is closed and exactly describable as that of a pseudo-qutrit.

## 3. Symmetries and Constants of Motion of ${\hat{H}}_{\mathrm{as}}$

It is easy to see that ${\hat{H}}_{as}$ is invariant with respect to the exchange of the homologous dynamic variables of the two qubits, that is, ${\hat{s}}_{1}\equiv {\hat{s}}_{1}⨂{\hat{I}}_{2}↔{\hat{I}}_{1}⨂{\hat{s}}_{2}\equiv {\hat{s}}_{2}$. Here, ${\hat{I}}_{1}$ and ${\hat{I}}_{2}$ are the identity operators in the Hilbert spaces of the first and second spin, respectively.

We now search for the existence of other symmetry transformations of ${\hat{H}}_{as}$, generated by substitutions of the six dynamic variables ${\hat{s}}_{1x}$, ${\hat{s}}_{1y}$, ${\hat{s}}_{1z}$, ${\hat{s}}_{2x}$, ${\hat{s}}_{2y}$, and ${\hat{s}}_{2z}$ with suitable operator expressions of such variables which satisfy all the pertinent commutation rules. We note that based on (13), the most general expression of all six dynamic variables that serves our purpose may only contain linear and bilinear terms, besides the 4×4 identity operator $\hat{I}$. The fulfillment of the exchange symmetry requires that the six substitutions be independent of ω, Ω, and θ. Therefore, each of them and all together must guarantee separate invariances of the six operators $\left({\hat{s}}_{1z}+{\hat{s}}_{2z}\right)$, ${\hat{s}}_{1x}{\hat{s}}_{2x}$, ${\hat{s}}_{1y}{\hat{s}}_{2y}$, ${\hat{s}}_{1z}{\hat{s}}_{2z}$, and $\left({\hat{s}}_{1z}{\hat{s}}_{2x}+{\hat{s}}_{1x}{\hat{s}}_{2z}\right)$ that appear in Equation (13).

By direct analytical construction, we found that ${\hat{H}}_{as}$ possesses a symmetry transformation which, to the best of our knowledge, has never been reported before. Since this transformation contains the free real parameter λ (vide infra), it indeed represents a class of symmetry and canonical transformations which, for a special value of the parameter λ, describes the exchange of the two spins (with respect to which ${\hat{H}}_{as}$ is invariant).

The canonical transformation leaving ${\hat{H}}_{as}$ invariant has the following form:

(16) $$\left\{\begin{array}{c} {\hat{s}}_{1i}\Rightarrow \frac{1+\cos\lambda }{2}{\hat{s}}_{1i}+\frac{1-\cos\lambda }{2}{\hat{s}}_{2i}-\sin\lambda \left({\hat{s}}_{1(i+2)}{\hat{s}}_{2(i+1)}-{\hat{s}}_{1(i+1)}{\hat{s}}_{2(i+2)}\right)\\ {\hat{s}}_{2i}\Rightarrow \frac{1-\cos\lambda }{2}{\hat{s}}_{1i}+\frac{1+\cos\lambda }{2}{\hat{s}}_{2i}+\sin\lambda \left({\hat{s}}_{1(i+2)}{\hat{s}}_{2(i+1)}-{\hat{s}}_{1(i+1)}{\hat{s}}_{2(i+2)}\right) \end{array}\right.$$

where i=x,y,z and λ is an arbitrary real parameter whose value can be limited to the range [0,2π]. If we imagine to arrange the symbols x,y,z in clockwise order at the vertices of an equilateral triangle, the subscript (i+r),r=1,2, then denotes the spin Cartesian component resulting after *r* clockwise jumps starting from component *i*. Accordingly, for example, if i=y, then y+1→z,y+2→x.

As we anticipated, it is remarkable that the class of λ-symmetry transformations in Equation (16) describes the exchange symmetry for λ=π. We do not report here the details of the analysis producing Equation (16) to avoid a lengthy mathematical digression, but it can be easily verified by direct substitution in ${\hat{H}}_{as}$ that the transformed operators (16) leave the Hamiltonian unchanged and satisfy the appropriate commutation rules.

The existence of a canonical symmetry transformation depending on a continuous parameter suggests that it could reflect the existence of a constant of motion in the dynamics of our system. To explore this idea, we will identify the unitary operator $\hat{V}$ that generates the transformation itself. For this purpose, it is worthy to note that the three Cartesian components of the total spin operator $\hat{S}={\hat{s}}_{1}+{\hat{s}}_{2}$ are invariant under the substitutions of Equation (16). This circumstance requires $\hat{V}$ to commute with ${\hat{S}}_{x}$, ${\hat{S}}_{y}$, and ${\hat{S}}_{z}$ separately. An operator acting in H commutes with these three operators if it is a function of ${\hat{S}}^{2}$. Thus, it is legitimate to seek a unitary operator $\hat{V}$ of the following form:

(17) $$\hat{V}=\exp\left(i\lambda {\hat{S}}^{2}\right)$$

This operator commutes neither with ${\hat{s}}_{1i}$ nor with ${\hat{s}}_{2i}$ for any i=x,y,z and generates six substitutions defined as follows:

(18) $$\left\{\begin{array}{c} {\hat{s}}_{1i}\Rightarrow \exp\left(-i\lambda {\hat{S}}^{2}\right){\hat{s}}_{1i}\exp\left(i\lambda {\hat{S}}^{2}\right)\\ {\hat{s}}_{2i}\Rightarrow \exp\left(-i\lambda {\hat{S}}^{2}\right){\hat{s}}_{2i}\exp\left(i\lambda {\hat{S}}^{2}\right) \end{array}\right.$$

It is possible to prove that these substitutions coincide with the ones we obtained earlier in Equation (16) following a different route, and that $\hat{V}$ is therefore the generator we were looking for.

It immediately follows that $\hat{V}$ commutes with ${\hat{H}}_{as}$ for any value of the continuous real parameter λ, and also that ${\hat{S}}^{2}$ itself is a constant of motion. This property can be verified directly, starting from the expression of the Hamiltonian. In fact, the commutation of ${\hat{S}}^{2}$ with ${\hat{H}}_{as}$ can be traced back to that between the Hamiltonian and the operator ${\hat{s}}_{1}{\hat{s}}_{2}$, which commutes with each of its three constitutive addends as well as with the operator $\left({\hat{s}}_{1z}{\hat{s}}_{2x}+{\hat{s}}_{1x}{\hat{s}}_{2z}\right)$, since different Cartesian components of a given spin 1/2 anticommute.

An important consequence of the conservation of ${\hat{S}}^{2}$ is that its nondegenerate eigenstate $|q_{A}\rangle =\frac{1}{\sqrt{2}}(|+\rangle |-\rangle -|-\rangle |+\rangle )$ is also an eigenstate of ${\hat{H}}_{as}$ with zero eigenvalue, whatever the θ value is. The antisymmetric state $|q_{A}\rangle$ is then the singlet eigenvector corresponding to the null eigenvalue of ${\hat{H}}_{as}$ that we predicted at the end of the previous section.

## 4. The Nested Qutrit Dynamics

The three-dimensional subspace $H_{S=1}$ of H, where ${\hat{S}}^{2}$ assumes its three-fold eigenvalue 2 = 1(1+1), is invariant for ${\hat{H}}_{as}$. Therefore, it is immediate to map the basis of the three eigenstates of $H_{S=1}$ into the basis of the three eigenstates associated with the three values of the *z*-component of a pseudo-qutrit $\hat{I}$ in its Hilbert space $H_{qt}$, namely:

(19) $$\left\{\begin{array}{c} |+\rangle |+\rangle \Rightarrow |1\rangle \\ |q_{S}\rangle =\frac{1}{\sqrt{2}}(|+\rangle |-\rangle +|-\rangle |+\rangle )\Rightarrow |0\rangle \\ |-\rangle |-\rangle \Rightarrow |-1\rangle \end{array}\right.$$

Therefore, the time evolution of our system of two qubits nests that of a pseudo-spin I = 1, henceforth simply named a qutrit, which means that when the initial state of our true system belongs to $H_{S=1}$, its evolution indeed simulates the qutrit dynamics in accordance with the mapping described by Equation (19).

To write the Hamiltonian for the qutrit, we need to change the matrix representation of ${\hat{H}}_{as}$ given in Equation (14), passing from the basis (15) to the following ordered basis of the eigenstates of ${\hat{S}}^{2}$:

(20) $$\begin{array}{ccc} |q_{1}\rangle & = & \left|+\rangle |+\rangle ,\;\right|q_{2}\rangle =|-\rangle |-\rangle ,\\ |q_{S}\rangle & = & \frac{1}{\sqrt{2}}(|+\rangle |-\rangle +|-\rangle |+\rangle ),\;|q_{A}\rangle =\frac{1}{\sqrt{2}}(|+\rangle |-\rangle -|-\rangle |+\rangle ). \end{array}$$

This alternative matrix representation of ${\hat{H}}_{as}$ is accomplished by the following unitary matrix:

(21) $$\hat{T}=\left(\begin{array}{cccc} 1 & 0 & 0 & 0\\ 0 & 1 & 0 & 0\\ 0 & 0 & 1/\sqrt{2} & -1/\sqrt{2}\\ 0 & 0 & 1/\sqrt{2} & 1/\sqrt{2} \end{array}\right),$$

since it leaves states $|\chi_{1}\rangle =|+\rangle |+\rangle$ and $\;|\chi_{2}\rangle =|-\rangle |-\rangle$ unchanged, while it clearly transforms $|\chi_{3}\rangle$ ($|\chi_{4}\rangle$) into $|q_{S}\rangle$ ($|q_{A}\rangle$).

Then, the transformed matrix assumes the following block form which reflects the invariance of ${\hat{H}}_{as}$ in $H_{S=1}$ (3×3 block) and in $H_{S=0}$ (1×1 block):

(22) $$\begin{array}{cc} \; & {\hat{\tilde{H}}}_{as}={\hat{T}}^{†}{\hat{H}}_{as}\hat{T}=\\ = & \frac{\Omega }{4}\left(\begin{array}{cccc} 1-3\cos^{2}\theta -x & -3\sin^{2}\theta & -\frac{3}{\sqrt{2}}\sin2\theta & 0\\ -3\sin^{2}\theta & 1-3\cos^{2}\theta +x & \frac{3}{\sqrt{2}}\sin2\theta & 0\\ -\frac{3}{\sqrt{2}}\sin2\theta & \frac{3}{\sqrt{2}}\sin2\theta & -2(1-3\cos^{2}\theta ) & 0\\ 0 & 0 & 0 & 0 \end{array}\right). \end{array}$$

where $x=\frac{4\omega }{\Omega }.$

We emphasize that Equation (22) represents a step toward the diagonalization of the Hamiltonian matrix (14) by a unitary transformation. This procedure was successfully carried out to solve a similar problem in [63] and further developed in [64]. In addition, this approach was applied to nonstationary problems in [65], where a new adiabatic representation was introduced.

On the basis of the mapping (19), the Hamiltonian ${\hat{H}}_{qt}$ of the qutrit, represented in its conventional ordered basis |+1〉, |0〉, |−1〉, can be obtained by making a permutation of the basis of the 3×3 block in $\hat{\tilde{H}}$, that is:

(23) $$\begin{array}{cc} {\hat{H}}_{qt}= & \frac{\Omega }{4}\left(\begin{array}{ccc} 1-3\cos^{2}\theta -x & -\frac{3}{\sqrt{2}}\sin2\theta & -3\sin^{2}\theta \\ -\frac{3}{\sqrt{2}}\sin2\theta & -2(1-3\cos^{2}\theta ) & \frac{3}{\sqrt{2}}\sin2\theta \\ -3\sin^{2}\theta & \frac{3}{\sqrt{2}}\sin2\theta & 1-3\cos^{2}\theta +x \end{array}\right). \end{array}$$

The Hamiltonian model that governs the dynamics of the qutrit can be given the following operator (and hence basis-independent) form:

(24) $${\hat{H}}_{qt}=\frac{\Omega }{4}\left(1-3\cos^{2}\theta \right)\left({\hat{I_{z}}}^{2}-\left({\hat{I_{x}}}^{2}+{\hat{I_{y}}}^{2}\right)\right)-\omega {\hat{I}}_{z}-\frac{3\Omega }{4}\sin2\theta \left(\hat{I_{z}}\hat{I_{x}}+\hat{I_{x}}\hat{I_{z}}\right)-\frac{3\Omega }{4}\sin\theta \left({\hat{I_{x}}}^{2}-{\hat{I_{y}}}^{2}\right),$$

where the operators for the components ${\hat{I}}_{x}$, ${\hat{I}}_{y}$, ${\hat{I}}_{z}$ of the qutrit $\hat{I}$ act in $H_{qt}$.

${\hat{H}}_{qt}$ could describe the interaction of a nucleus that has spin 1 and an electric quadrupole moment (such as ${}^{14}N$, for example) subjected to a magnetic field and exposed to an external inhomogeneous electric field. It is a remarkable consequence of the axial symmetry of the hyperfine tensor (12) considered in this paper that the dynamic behavior of the qutrit $\hat{I}$ can be simulated by resorting to the dipolar interaction of two qubits.

When the initial state of the two qubits is represented by any density operator ρ such that $Tr(\rho {\hat{S}}^{2})=2$, the last row and the last column of the matrix representing it in the basis (20) do not evolve over time. On the contrary, the remaining 3×3 block has a time evolution, and according to our results and mapping (19), simulates the time dependence of the qutrit determined by the Liouville equation:

(25) $$\frac{\partial \rho^{\left(1\right)}}{\partial t}+i\left[{\hat{H}}_{qt},\rho^{\left(1\right)}\right]=0$$

in the Hilbert space $H_{qt}$, where $\rho^{\left(1\right)}$ is the density matrix of the qutrit described by the Hamiltonian model (24). It is worth noting that Equation (25) also holds when the applied magnetic field is time-dependent. To appreciate this fact, it is sufficient to consider that the canonical transformation (21) does not involve ω. This circumstance implies that the Liouville equation of the two-spin system retains its formal structure (that is, no additional term is produced by the transformation *T*), where ${\hat{H}}_{as}$ is clearly changed to ${\hat{\tilde{H}}}_{as}$. By virtue of the block form of the matrix representing ${\hat{\tilde{H}}}_{as}$, the transformed Liouville equation for the two spins generates (25). Solving this equation when ω depends on time is out of the scope of this study. In the next section, therefore, we will continue to consider the application of static magnetic fields.

## 5. Eigenvalues of ${\hat{H}}_{\mathrm{qt}}$ (and of ${\hat{H}}_{\mathrm{as}}$)

By examining the elements of the matrix (23), one can see that the characteristic equation of $\frac{4}{\Omega }{\hat{H}}_{qt}$ can be cast in the following dimensionless reduced ($tr{\hat{H}}_{qt}=0$) form:

(26) $$\eta^{3}-p\eta +q=0,$$

where p≡p(x) and q≡q(x,θ) are both quadratic even polynomials with respect to *x*, that is:

(27) $$\left\{\begin{array}{c} p=12+x^{2}>0\\ q=q_{0}\left(\cos^{2}\theta \right)+q_{2}\left(\cos^{2}\theta \right)x^{2}. \end{array}\right.$$

It can easily be verified that $q_{0}\left(\cos^{2}\theta \right)$ and $q_{2}\left(\cos^{2}\theta \right)$ have the following expressions:

(28) $$\left\{\begin{array}{c} q_{0}\left(\cos^{2}\theta \right)=216\left(\cos^{6}\theta -2\cos^{4}\theta +\cos^{2}\theta \right)-16\\ q_{2}\left(\cos^{2}\theta \right)=-2\left(1-3\cos^{2}\theta \right). \end{array}\right.$$

The dependence of the coefficient *q* in Equation (26) on θ through $\cos^{2}\theta$ is remarkable because it means that the eigenvalues of ${\hat{H}}_{qt}$ (as well as of ${\hat{H}}_{as}$) coincide in correspondence to the two supplementary inclinations θ and (π−θ) of the hyperfine tensor symmetry axis defined in Equation (12).

The three real roots of Equation (26) can be expressed in trigonometric form as follows:

(29) $$\eta_{k}=2\sqrt{\frac{p}{3}}\cos\frac{\phi +2k\pi }{3},\;k=0,1,2$$

where

(30) $$\phi =\arccos\frac{3q}{2p}\sqrt{\frac{p}{3}}.$$

The three eigenvalues of ${\hat{H}}_{qt}$ related to the three roots of Equation (26) given by Equation (29) are obviously $\varepsilon_{k}=\frac{\Omega }{4}\eta_{k}$, and, for a generic value of θ, their expressions are algebraically inconvenient to handle, mainly due to the dependence of the coefficient *q* appearing in Equation (26) on θ. In some particular cases, however, the following observation makes it possible to write the roots of Equation (26) in a relatively simpler way. The dependence of the coefficients *p* and *q* on *x*, as given in Equations (27) and (28), suggests a necessary condition for the existence of a root of Equation (26) independent of *x*. Equations (27) and (28) show that if such a root exists at particular values of θ, it must evidently fulfill the condition below:

(31) $$\eta =-2(1-3\cos^{2}\theta ).$$

To find all the θ values for which the previous observation actually allows us to determine a root of (26), we proceed by direct substitution of the test root (31) in Equation (26), thus obtaining the following condition on $z=\cos^{2}\theta$:

(32) $$2z^{3}-3z^{2}+z=0,$$

which, in [0,π), clearly has the following roots:

(33) $$\left\{\begin{array}{c} \cos^{2}\theta =0\to \theta =\frac{\pi }{2},\eta =-2\\ \cos^{2}\theta =1\to \theta =0,\eta =4\\ \cos^{2}\theta =\frac{1}{2}\to \theta =\frac{\pi }{4},\frac{3\pi }{4},\eta =1, \end{array}\right.$$

In Equation (33), we have also indicated the corresponding acceptable test roots.

The first and second roots correspond to the physical situations in which the magnetic field is orthogonal and parallel to the hyperfine tensor axis, respectively. In the third case, the axis is instead tilted by $\frac{\pi }{4}$ or $\frac{3\pi }{4}$ with respect to the field direction. We point up that these are the only cases in which the test roots help to straightforwardly find the three eigenvalues of ${\hat{H}}_{qt}$. In all other geometric situations, its eigenvalues must be deduced using Equation (29) and the related Equation (30).

We can write down the three eigenvalues of ${\hat{H}}_{qt}$ for each of the three cases successfully solved with the help of the test roots (32):

(34) $$\left\{\begin{array}{c} \theta =0\to \Omega ,\frac{\Omega }{2}+\omega ,\frac{\Omega }{2}-\omega \\ \theta =\frac{\pi }{2}\to -\frac{\Omega }{2},\frac{\Omega }{4}+\sqrt{{\left(\frac{3\Omega }{4}\right)}^{2}+\omega^{2}},\frac{\Omega }{4}-\sqrt{{\left(\frac{3\Omega }{4}\right)}^{2}+\omega^{2}}\\ \theta =\frac{\pi }{4}\to \frac{\Omega }{4},\frac{\Omega }{8}\left(-1-\sqrt{45+4x^{2}}\right),\frac{\Omega }{8}\left(-1+\sqrt{45+4x^{2}}\right). \end{array}\right.$$

The matrix (23) has different elements when θ changes from $\frac{\pi }{4}$ to $\frac{3\pi }{4}$. Nevertheless, the matrices corresponding to these two values of θ are related by a unitary transformation, as they are both diagonalizable and their eigenvalues coincide.

The knowledge of the exact eigenvalues of ${\hat{H}}_{as}$ enables the evaluation of the corresponding eigenvectors, and thus the investigation of the dynamics of the two-spin system, as well as that of the nested qutrit; however, in the rest of this study, we will turn our attention to general connections between the properties exhibited by the density matrix of the two spins and that of the emerged qutrit.

## 6. Representation of the Density Matrix in the Qutrit Subspace

The standard basis set (2) corresponds to a direct product of two subsystem states. The basis set (15) is derived from a permutation of the standard one. If we compactly describe a basis set as a four-dimension vector $\vec{\chi }$ and a standard basis set as $\vec{m}$, this permutation can be represented by a 4×4 matrix $\hat{P}:\;\vec{\chi }=\hat{P}\vec{m}$, where matrix $\hat{P}$ is defined by the following equation:

(35) $$\left(\begin{array}{c} |\chi_{1}\rangle \\ |\chi_{2}\rangle \\ |\chi_{3}\rangle \\ |\chi_{4}\rangle \end{array}\right)=\left(\begin{array}{cccc} 1 & 0 & 0 & 0\\ 0 & 0 & 0 & 1\\ 0 & 0 & 1 & 0\\ 0 & 1 & 0 & 0 \end{array}\right)\left(\begin{array}{c} |+\rangle |+\rangle \\ |+\rangle |-\rangle \\ |-\rangle |+\rangle \\ |-\rangle |-\rangle \end{array}\right)$$

Let us represent the generic density matrix $\rho_{s}^{\left(1/2\times 1/2\right)}$ of our two-spin system in the standard basis as follows:

(36) $$\rho_{s}^{\left(1/2\times 1/2\right)}=\left(\begin{array}{cccc} R_{1} & R_{5} & R_{6} & R_{7}\\ {R_{5}}^{*} & R_{2} & R_{8} & R_{9}\\ {R_{6}}^{*} & {R_{8}}^{*} & R_{3} & R_{10}\\ {R_{7}}^{*} & {R_{9}}^{*} & {R_{10}}^{*} & R_{4} \end{array}\right),\;R_{1}+R_{2}+R_{3}+R_{4}=1.$$

The unitary operator PT accomplishes the transformation of $\rho_{s}^{\left(1/2\times 1/2\right)}$ from the representation in the standard basis to the representation in the entangled basis (20) of the eigenstates of $S^{2}$.

The new representation of the density matrix, denoted by $\rho^{\left(1,0\right)}$, can be formally written as follows:

(37) $$\rho^{\left(1,0\right)}\equiv {\hat{T}}^{†}{\hat{P}}^{†}\rho_{s}^{\left(1/2\times 1/2\right)}\hat{P}\hat{T}=\left(\begin{array}{cccc} r_{1} & r_{4} & r_{5} & \beta \\ {r_{4}}^{*} & r_{2} & r_{6} & \gamma \\ {r_{5}}^{*} & {r_{6}}^{*} & r_{3} & \delta \\ \beta^{*} & \gamma^{*} & \delta^{*} & \alpha \end{array}\right).$$

where $r_{1}+r_{2}+r_{3}+\alpha =1$. If the two-spin system is initially prepared in the state (37), with no restrictions on the the elements α,β, and γ, the initial mean value of $S^{2}$ is positive and generally less than 2 and remains constant during the time evolution of the binary system. Since we are interested in evidencing the emergence of the subdynamics of the nested qutrit, we confine ourselves to a special sub-class of the density matrices $\rho^{\left(1,0\right)}$:

(38) $$\rho^{\left(1,0\right)}\equiv {\hat{T}}^{†}{\hat{P}}^{†}\rho_{s}^{\left(1/2\times 1/2\right)}\hat{P}\hat{T}=\left(\begin{array}{cccc} r_{1} & r_{4} & r_{5} & 0\\ {r_{4}}^{*} & r_{2} & r_{6} & 0\\ {r_{5}}^{*} & {r_{6}}^{*} & r_{3} & 0\\ 0 & 0 & 0 & 0 \end{array}\right),$$

namely that in which the elements of the fourth row and column vanish at t=0 and then at any successive time. We remark that under such a restriction, the 3×3 block appearing in (38) can be interpreted as the initial density matrix of the nested qutrit.

Since the matrix (36) is the most common way of representing the initial density matrix of the two-spin system, we seek the conditions to be imposed on its elements so that its representation in the entangled basis (20) transparently exhibits the emergence of the nested qutrit, that is, assumes the form (38).

Acting on the density matrix (36) as prescribed in (37) and requiring that the resulting matrix has the form (38) yield the following conditions on the elements of $\rho_{s}^{\left(1/2\times 1/2\right)}$:

(39) $$R_{2}=R_{3},\;R_{5}=R_{6},\;R_{9}=R_{10},\;R_{8}=\frac{1}{2}\left(R_{2}+R_{3}\right).$$

Furthermore, we obtain the expressions of the six independent matrix elements:

(40) $$r_{1}=R_{1},\;r_{2}=R_{4},\;r_{3}=2R_{2},\;r_{4}=R_{7},\;r_{5}=\sqrt{2}R_{5},\;r_{6}=2\sqrt{2}{R_{9}}^{*}.$$

We emphasize that Equation (39) provides the recipe for writing an initial density matrix of two spins whose time evolution, in accordance with the mapping (19), simulates the dynamics of a qutrit with an initial density matrix given by the 3×3 block of (38), where the elements satisfy Equation (40). Therefore, we have the necessary and sufficient conditions for the emergence of a qutrit dynamics in the dipolar coupling of two qubits. At the same time, we are legitimated to claim that preparing the two spins in a generic state does not generally lead to the same dynamic behavior, which means that the invariant subspaces of $S^{2}$ are generally mixed at t=0.

To better appreciate the above statement and exemplify its feasibility, it is interesting to investigate the compatibility between the two representations (36) and (38) when the former describes the simplest separable (factorable) state of the two-spin system, namely $\rho_{s}^{\left(1/2\times 1/2\right)}=\rho_{1}^{\left(1/2\right)}⊗\rho_{2}^{\left(1/2\right)}$.

To this end, exploiting the well-known general characterization of the density matrix of a qubit by a polarization vector P, we first write the following equation:

(41) $$\rho_{s}^{\left(1/2\times 1/2\right)}=\rho_{1}^{\left(1/2\right)}⊗\rho_{2}^{\left(1/2\right)}=\frac{1}{4}\left(1+P^{\left(1\right)}\sigma_{1}\right)⊗\left(1+P^{\left(2\right)}\sigma_{2}\right),$$

where $\sigma_{i}$ and $P^{\left(i\right)}\equiv tr\left(\sigma_{i}\rho_{i}^{1/2}\right)$ are the Pauli matrices and the polarization vector for the i-th spin, respectively. After simple algebra, we find the following relations between the components of the vectors $P^{\left(1\right)}$ and $P^{\left(2\right)}$ and the matrix elements (36):

(42) $$\begin{array}{cc} R_{1}= & \frac{1}{4}\left(1+P_{z}^{\left(1\right)}\right)\left(1+P_{z}^{\left(2\right)}\right),\;R_{2}=\frac{1}{4}\left(1+P_{z}^{\left(1\right)}\right)\left(1-P_{z}^{\left(2\right)}\right),\\ R_{3}= & \frac{1}{4}\left(1-P_{z}^{\left(1\right)}\right)\left(1+P_{z}^{\left(2\right)}\right),\;R_{4}=\frac{1}{4}\left(1-P_{z}^{\left(1\right)}\right)\left(1-P_{z}^{\left(2\right)}\right),\\ R_{5}= & \frac{1}{4}\left(1+P_{z}^{\left(1\right)}\right)P_{-}^{\left(2\right)},\;R_{6}=\frac{1}{4}P_{-}^{\left(1\right)}\left(1+P_{z}^{\left(2\right)}\right),\;R_{7}=\frac{1}{4}P_{-}^{\left(1\right)}P_{-}^{\left(2\right)},\\ R_{8}= & \frac{1}{4}P_{-}^{\left(1\right)}P_{+}^{\left(2\right)},\;R_{9}=\frac{1}{4}P_{-}^{\left(1\right)}\left(1-P_{z}^{\left(2\right)}\right),\;R_{10}=\frac{1}{4}\left(1-P_{z}^{\left(1\right)}\right)P_{-}^{\left(2\right)}. \end{array}$$

where $P_{\pm }^{\left(a\right)}=P_{x}^{\left(a\right)}\pm iP_{y}^{\left(a\right)}$.

We now impose the conditions (39). Since $R_{8}$ is real, we also obtain that:

(43) $$P_{x}^{\left(1\right)}P_{y}^{\left(2\right)}=P_{y}^{\left(1\right)}P_{x}^{\left(2\right)},\;or\;\frac{P_{x}^{\left(1\right)}}{P_{y}^{\left(1\right)}}\;=\;\frac{P_{x}^{\left(2\right)}}{P_{y}^{\left(2\right)}}.$$

The relations $R_{5}=R_{6}$ and $R_{9}=R_{10}$ are equivalent to Equation (43) and therefore cannot be regarded as independent. The equality $R_{2}=R_{3}$ requires that $P_{z}^{\left(1\right)}=P_{z}^{\left(2\right)}$, thus implying a restriction on the transverse components, namely $P_{-}^{\left(1\right)}=P_{-}^{\left(2\right)}$, or $P_{x}^{\left(1\right)}=P_{x}^{\left(2\right)}$ and $P_{y}^{\left(1\right)}\;=\;P_{y}^{\left(2\right)}$.

We thus necessarily obtain the following:

(44) $$P^{\left(1\right)}=P^{\left(2\right)}=P.$$

Excluding (based on our defined goal) the mixed state of a singlet and a triplet in the class of density matrices (38), we obtain the additional condition:

(45) $$P^{2}=1.$$

Since, for any qubit state, $P^{2}\equiv <\sigma {>}^{2}\leq 1$, the strong constraint (45) means that the only separable density matrix (41) of the two spins compatible with the emergence of a qutrit is the product of two homologous eigenstates of any spin component.

It is now instructive to analyze the same problem under scrutiny in this section in terms of classical probabilities.

Let $p_{1},\;p_{2}$, and $p_{3}$ be the classical probabilities to measure positive spin-1/2 projections on the axes x,y, and *z*, respectively. As was shown in [66], the density matrix can be written as follows:

(46) $$\rho^{\left(p\right)}=\left(\begin{array}{cc} p_{3} & p_{-}-e^{-i\pi /4}/\sqrt{2}\\ p_{+}-e^{i\pi /4}/\sqrt{2} & 1-p_{3} \end{array}\right),\;where\;p_{\pm }=p_{1}\pm ip_{2}.$$

We can represent the density matrix of the two spins 1/2 at the initial time as the direct product of two density matrices determined by Equation (46):

(47) $$\begin{array}{cc} \; & \rho_{s}^{\left(1/2\times 1/2\right)}=\rho_{1}^{\left(p\right)}⊗\rho_{2}^{\left(p\right)}=\\ \; & =\left(\begin{array}{cc} p_{3}^{\left(1\right)} & p_{-}^{\left(1\right)}-e^{-i\pi /4}/\sqrt{2}\\ p_{+}^{\left(1\right)}-e^{i\pi /4}/\sqrt{2} & 1-p_{3}^{\left(1\right)} \end{array}\right)⊗\left(\begin{array}{cc} p_{3}^{\left(2\right)} & p_{-}^{\left(2\right)}-e^{-i\pi /4}/\sqrt{2}\\ p_{+}^{\left(2\right)}-e^{i\pi /4}/\sqrt{2} & 1-p_{3}^{\left(2\right)} \end{array}\right). \end{array}$$

We need to verify the relations in the system of Equation (39) for the composite density matrix (47). As in (42), we have the following relations:

$$\begin{array}{cc} R_{1} & =p_{3}^{\left(1\right)}p_{3}^{\left(2\right)},\;R_{2}=p_{3}^{\left(1\right)}\left(1-p_{3}^{\left(2\right)}\right),\;R_{3}=\left(1-p_{3}^{\left(1\right)}\right)p_{3}^{\left(2\right)},\;R_{4}=\left(1-p_{3}^{\left(1\right)}\right)\left(1-p_{3}^{\left(2\right)}\right), \end{array}$$

(48) $$\begin{array}{cc} R_{7} & =\left(p_{-}^{\left(1\right)}-e^{-i\pi /4}/\sqrt{2}\right)\left(p_{-}^{\left(2\right)}-e^{-i\pi /4}/\sqrt{2}\right),\;R_{8}=\left(p_{-}^{\left(1\right)}-e^{-i\pi /4}/\sqrt{2}\right)\left(p_{+}^{\left(2\right)}-e^{i\pi /4}/\sqrt{2}\right)\\ R_{5} & =p_{3}^{\left(1\right)}\left(p_{-}^{\left(2\right)}-\frac{e^{-i\pi /4}}{\sqrt{2}}\right),\;R_{6}=\left(p_{-}^{\left(1\right)}-\frac{e^{-i\pi /4}}{\sqrt{2}}\right)p_{3}^{\left(2\right)}, \end{array}$$

(49) $$\begin{array}{cc} R_{9} & =\left(p_{-}^{\left(1\right)}-\frac{e^{-i\pi /4}}{\sqrt{2}}\right)\left(1-p_{3}^{\left(2\right)}\right),\;R_{10}=\left(1-p_{3}^{\left(1\right)}\right)\left(p_{-}^{\left(2\right)}-\frac{e^{-i\pi /4}}{\sqrt{2}}\right). \end{array}$$

Using the condition $ℑR_{8}=0$, we obtain a relation similar to Equation (43):

(50) $$\left(p_{1}^{\left(1\right)}-\frac{1}{2}\right)\left(p_{2}^{\left(2\right)}-\frac{1}{2}\right)=\left(p_{2}^{\left(1\right)}-\frac{1}{2}\right)\left(p_{1}^{\left(2\right)}-\frac{1}{2}\right).$$

The condition $R_{2}=R_{3}$ implies the following relation:

(51) $$p_{3}^{\left(1\right)}\left(1-p_{3}^{\left(2\right)}\right)=\left(1-p_{3}^{\left(1\right)}\right)p_{3}^{\left(2\right)}\;\Rightarrow \;p_{3}^{\left(1\right)}=p_{3}^{\left(2\right)}.$$

Now, we can see that

$$\left(p_{3}^{\left(1\right)}-\frac{1}{2}\right)\left(p_{3}^{\left(2\right)}-\frac{1}{2}\right)=-\frac{1}{2}p_{3}^{\left(1\right)}\left(1-p_{3}^{\left(2\right)}\right)-\frac{1}{2}p_{3}^{\left(2\right)}\left(1-p_{3}^{\left(1\right)}\right)+\frac{1}{4}=-R_{2}+\frac{1}{4}.$$

Moreover,

$$ℜR_{8}=\left(p_{1}^{\left(1\right)}-\frac{1}{2}\right)\left(p_{1}^{\left(2\right)}-\frac{1}{2}\right)+\left(p_{2}^{\left(1\right)}-\frac{1}{2}\right)\left(p_{2}^{\left(2\right)}-\frac{1}{2}\right).$$

Let us introduce a “shifted” probability vector as [66]:

(52) $${\vec{p}{\;}^{\left(i\right)}}^{\prime }=\left(p_{1}^{\left(i\right)}-\frac{1}{2},\;p_{2}^{\left(i\right)}-\frac{1}{2},\;p_{3}^{\left(i\right)}-\frac{1}{2}\right).$$

With this notation, relation (39) is written as follows:

(53) $$\left({\vec{p}{\;}^{\left(1\right)}}^{\prime },{\vec{p}{\;}^{\left(2\right)}}^{\prime }\right)=\frac{1}{4}.$$

We also notice some constraints on the probabilities $p_{1},\;p_{2}$, and $p_{3}$ that result from the positive definiteness of density matrices [66]. The simplest way to obtain these constraints is to calculate $\det\rho^{\left(p\right)}$ for the density matrix (46):

(54) $$\det\rho^{\left(p\right)}⩾0\;or\;\left(\vec{p}{\;}^{\prime },\vec{p}{\;}^{\prime }\right)⩽\frac{1}{4}.$$

We can therefore consider the equality (53) as a realization of the inequality (54) for two identical qubits in state (46) at t=0. Furthermore, the relation (53) can be treated as the Born rule for two qubits [66,67,68].

The relations in (39) reduce to the following equations:

(55) $$\begin{array}{cc} R_{5}=R_{6}\; & \to \;p_{3}^{\left(1\right)}\left(p_{-}^{\left(2\right)}-\frac{e^{-i\pi /4}}{\sqrt{2}}\right)=\left(p_{-}^{\left(1\right)}-\frac{e^{-i\pi /4}}{\sqrt{2}}\right)p_{3}^{\left(2\right)}, \end{array}$$

(56) $$\begin{array}{cc} R_{9}=R_{10}\; & \to \;\left(p_{-}^{\left(1\right)}-\frac{e^{-i\pi /4}}{\sqrt{2}}\right)\left(1-p_{3}^{\left(2\right)}\right)=\left(1-p_{3}^{\left(1\right)}\right)\left(p_{-}^{\left(2\right)}-\frac{e^{-i\pi /4}}{\sqrt{2}}\right). \end{array}$$

Equation (56) gives

(57) $$p_{-}^{\left(1\right)}=p_{-}^{\left(2\right)}.$$

Finally, we formulate relations for the probability vectors similar to those for the polarizations:

(58) $${\vec{p}{\;}^{\prime }}^{\left(1\right)}={\vec{p}{\;}^{\prime }}^{\left(2\right)}=\vec{p}{\;}^{\prime }.$$

The results obtained closely match those obtained above in terms of polarization vectors; that is, the two identical particles should have equal probability vectors to satisfy the necessary conditions for separability. A qutrit state can only be formed in this case.

## 7. Qutrit State Measurement

We can measure qutrit states if we connect them with physical observables, and we can begin with the spin polarization. For the longitudinal component (we use the fact that $R_{2}=R_{3}$), we can write the following equation:

(59) $$P_{z}^{\left(1\right)}=\mathrm{Tr}\sigma_{z}^{\left(1\right)}\rho =R_{1}+R_{2}-R_{3}-R_{4}=r_{1}\left(t\right)-r_{2}\left(t\right),$$

and for the transverse component:

(60) $$\;P_{+}^{\left(1\right)}=\mathrm{Tr}\sigma_{+}^{\left(1\right)}\rho =\sqrt{2}\left({r_{5}}^{*}\left(t\right)+r_{6}\left(t\right)\right).$$

Based on Equation (44), it can easily be seen that:

(61) $$P_{z}^{\left(1\right)}\left(t\right)=P_{z}^{\left(2\right)}\left(t\right)=P_{z}\left(t\right),\;and\;P_{+}^{\left(1\right)}\left(t\right)=P_{+}^{\left(2\right)}\left(t\right)=P_{+}\left(t\right).$$

In the following, we will consider two situations in which a static external magnetic field B∥n (θ=0) and B⊥n (θ=π/2). In the first case, the problem is simplified by the fact that the Hamiltonian matrix can be easily diagonalized. The transition frequencies are determined by Equation (34) and are equal to

(62) $$\omega_{13}=-\frac{3}{2}\Omega -\omega ,\;\omega_{23}=-\frac{3}{2}\Omega +\omega .$$

Assuming Equation (41) as the initial density matrix of the two qubits, we have

(63) $$\begin{array}{cc} P_{+}\left(t\right)= & \frac{1}{4}\left(\left(1+P_{z}\left(0\right)\right)e^{i\omega_{13}t}+\left(1-P_{z}\left(0\right)\right)e^{-i\omega_{23}t}\right)P_{+}\left(0\right)\\ = & \frac{1}{2}\left(\cos\frac{3\Omega }{2}t-iP_{z}\left(0\right)\sin\frac{3\Omega }{2}t\right)e^{-i\omega t}P_{+}\left(0\right). \end{array}$$

where we used the relation $P^{\left(1\right)}\left(0\right)=P^{\left(2\right)}\left(0\right)$.

When the representation (47) in terms of classical probabilities is used instead, we obtain

(64) $$\begin{array}{cc} P_{+}\left(t\right)= & 2\left(p_{+}-\frac{e^{i\pi /4}}{\sqrt{2}}\right)\left(p_{3}e^{i\omega_{13}t}+\left(1-p_{3}\right)e^{-i\omega_{23}t}\right)\\ = & 2\left(p_{+}-\frac{e^{i\pi /4}}{\sqrt{2}}\right)\left(p_{3}\cos\frac{3\Omega }{2}t-i\left(2p_{3}-1\right)\sin\frac{3\Omega }{2}t\right)e^{-i\omega t}. \end{array}$$

Equation (58) was used here.

When B⊥n, the Hamiltonian (14) consists of two independent blocks, each being of dimension two. The 2 × 2 Hamiltonian of the first block acts on the subspace spanned by the states $|q_{1}\rangle$ and $|q_{2}\rangle$ defined in (20) and can be represented as the effective operator:

(65) $${\hat{H}}_{12}=\frac{\Omega }{4}-\omega \sigma_{z}-\frac{3}{4}\Omega \sigma_{x},$$

where $\sigma_{z}$ and $\sigma_{x}$ are Pauli matrices. Then, we can derive the evolution operator as follows:

(66) $$\begin{array}{cc} U_{12}\left(t\right)= & \exp\left[-i\left(\frac{\Omega }{4}-\omega \sigma_{z}-\frac{3}{4}\Omega \sigma_{x}\right)t\right]=\\ = & e^{-i\Omega t/4}\left(\cos\tilde{\Omega }t+i\left(\frac{\omega }{\tilde{\Omega }}\sigma_{z}+\frac{3\Omega }{4\tilde{\Omega }}\sigma_{x}\right)\sin\tilde{\Omega }t\right), \end{array}$$

where $\tilde{\Omega }=\sqrt{{\left(3\Omega /4\right)}^{2}+\omega^{2}}$.

Compared to the first case, in which θ=0, the longitudinal polarization contains oscillating terms. This effect is very pronounced and lends itself to experimental observation. The transverse component of the polarization is rather cumbersome, and we will not present it here.

Equation (59) shows that in order to calculate the longitudinal polarization we only need to determine the two diagonal elements, $r_{1}\left(t\right)$ and $r_{2}\left(t\right)$, of the qutrit density matrix:

$$P_{z}^{\left(1\right)}\left(t\right)=P_{z}^{\left(2\right)}\left(t\right)\equiv P_{z}\left(t\right)=r_{1}\left(t\right)-r_{2}\left(t\right).$$

After some lengthy algebra, we obtain the following:

(67) $$\begin{array}{cc} P_{z}\left(t\right)= & \left[\cos^{2}\tilde{\Omega }t+\left({\left(\frac{\omega }{\tilde{\Omega }}\right)}^{2}-{\left(\frac{3\Omega }{4\tilde{\Omega }}\right)}^{2}\right)\sin^{2}\tilde{\Omega }t\right]\left(r_{1}\left(0\right)-r_{2}\left(0\right)\right)+\\ + & 6\frac{\Omega }{\tilde{\Omega }}\sin\tilde{\Omega }tℑ\left[\left(\cos\tilde{\Omega }t+i\frac{\omega }{\tilde{\Omega }}\sin\tilde{\Omega }t\right)r_{4}\left(0\right)\right]. \end{array}$$

Next, we will show the results for the two different representations of the density matrix. In the representation in terms of polarization vectors, we have $R_{1}={\left(1+P_{z}\right)}^{2}/4$ and $R_{4}={\left(1-P_{z}\right)}^{2}/4$, and hence $r_{1}\left(0\right)-r_{2}\left(0\right)=R_{1}-R_{4}=P_{z}\left(0\right)$. For the matrix element $r_{4}\left(0\right)$, we obtain $r_{4}=R_{7}=P_{-}^{2}/4=\left(P_{x}^{2}-P_{y}^{2}\right)/4+iP_{x}P_{y}/2$. The longitudinal polarization is described by the equation below:

(68) $$P_{z}\left(t\right)=\left[{\left(\frac{3\Omega }{4\tilde{\Omega }}\right)}^{2}\cos2\tilde{\Omega }t+{\left(\frac{\omega }{\tilde{\Omega }}\right)}^{2}\right]P_{z}\left(0\right)-\frac{3\Omega }{2\tilde{\Omega }}\sin2\tilde{\Omega }tP_{x}P_{y}-\frac{3\Omega \omega }{4{\tilde{\Omega }}^{2}}\left(1-\cos2\tilde{\Omega }t\right)\left(P_{x}^{2}-P_{y}^{2}\right).$$

which yields a simple and well-known behavior when P(0)∥z.

Using the probability representation, the use of Equation (59) leads to the following relations:

(69) $$\begin{array}{cc} \; & r_{1}\left(0\right)-r_{2}\left(0\right)=2p_{3}-1,\\ \; & r_{4}=R_{7}={\left(p_{+}-\frac{e^{i\pi /4}}{\sqrt{2}}\right)}^{2}={\left(p_{1}-\frac{1}{2}\right)}^{2}-{\left(p_{2}-\frac{1}{2}\right)}^{2}+i2\left(p_{1}-\frac{1}{2}\right)\left(p_{2}-\frac{1}{2}\right). \end{array}$$

The longitudinal polarization is then determined by Equation (68) changing $P_{x}\to p_{1}-1/2$ and $P_{y}\to p_{2}-1/2$.

## 8. Conclusions

In this study, we have brought to light the emergence of the quantum dynamics of a pseudo-qutrit in the time evolution of two identical but distinguishable spins 1/2, which are dipolarly coupled and subjected to an external static and uniform magnetic field of arbitrary intensity. The Hamiltonian model leading to this conclusion was extracted from the general model that describes the dynamics of the spin system by introducing a symmetry constraint in the physical scenario. The constraint consists in the requirement that the hyperfine tensor be axially symmetric. Among the various ways to satisfy this requirement, we chose the one in which n is parallel to the xz plane. The corresponding specialized Hamiltonian model can be investigated in a more profitable way, and the existence of a novel, nonlinear, canonical symmetry transformation was revealed. This symmetry transformation is generated by ${\hat{S}}^{2}$ and includes the invariance of the specialized Hamiltonian model under the exchange of the two spins. We remark that other ways of making A axially symmetric exist, since there is no reason why the condition $n_{2}=0$ should play a special role. In fact, by fixing the matrix A as a two-block matrix, one obtains either $n_{1}=0$ or $n_{3}=0$. It is worth noting that no qualitative difference in the results reported in this study would appear starting from a different choice of the form of matrix A. We emphasize that, given a system of two dipolarly coupled distinguibisgable qubits acted upon by a uniform and static magnetic field, the control of the orientation of the binary system with respect to the laboratory frame is within the reach of the experimentalists [61]. Therefore, the emergence or disappearance of pseudo-qutrit quantum dynamics could be investigated experimentally.

The paper also presents two systematic approaches that highlight the link between the initial density matrix of the two spins and that of the pseudo qutrit. The first approach explores the advantages stemming from the description of the state of the two spins in terms of polarization vectors. The second approach is instead based on the use of classical probabilities to describe the initial state of the two spins. Both approaches suggest that a qutrit state can be observed by measuring the spin polarization of the system for two different orientations of a static external magnetic field, with relevance to quantum information and quantum computing studies. In fact, formulas (64) and (68) show how the parameters involved in the probability representation can be obtained by measuring the longitudinal and transverse spin polarizations.

The density matrix for a qutrit state of two identical spin-1/2 systems can also be described in the probability representation [16]. Then, the probabilities that determine the qutrit density matrix could be expressed in terms of the probabilities determining the spin-1/2 density matrix. We will discuss this possibility in a future study.

## Data Availability

All necessary data is contained in the article.
